# Comparative Evaluation of Inducible Cre Mouse Models for Fibroblast Targeting in the Healthy and Infarcted Myocardium

**DOI:** 10.3390/biomedicines10102350

**Published:** 2022-09-21

**Authors:** Laura Pilar Aguado-Alvaro, Nerea Garitano, Gloria Abizanda, Eduardo Larequi, Felipe Prosper, Beatriz Pelacho

**Affiliations:** 1 Regenerative Medicine Department, Center for Applied Medical Research (CIMA)-Universidad de Navarra, 31008 Pamplona, Spain; 2 Instituto de Investigación Sanitaria de Navarra (IdiSNA), 31008 Pamplona, Spain; 3 Department of Cell Therapy and Hematology, Clínica Universidad de Navarra, 31008 Pamplona, Spain

**Keywords:** cardiac fibroblast, myocardial infarction, animal model, Cre recombinase

## Abstract

Several Cre recombinase transgenic mouse models have been generated for cardiac fibroblast (CF) tracking and heart regulation. However, there is still no consensus on the ideal mouse model to optimally identify and/or regulate these cells. Here, a comparative evaluation of the efficiency and specificity of the indirect reporter Cre-loxP system was carried out in three of the most commonly used fibroblast reporter transgenic mice (Pdgfr*a*-CreERT2, Col1a1-CreERT2 and PostnMCM) under healthy and ischemic conditions, to determine their suitability in in vivo studies of cardiac fibrosis. We demonstrate optimal Cre recombinase activity in CF (but also, although moderate, in endothelial cells (ECs)) derived from healthy and infarcted hearts in the PDGFR*a*-creERT2 mouse strain. In contrast, no positive reporter signal was found in CF derived from the Col1a1-CreERT2 mice. Finally, in the PostnMCM line, fluorescent reporter expression was specifically detected in activated CF but not in EC, which leads us to conclude that it may be the most reliable model for future studies on cardiovascular disease. Importantly, no lethality or cardiac fibrosis were induced after tamoxifen administration at the established doses, either in healthy or infarcted mice of the three fibroblast reporter lineages. This study lays the groundwork for future efficient in vivo CF tracking and functional analyses.

## 1. Introduction

Cardiovascular diseases are the leading cause of morbidity and mortality worldwide, with myocardial infarction (MI) and subsequent heart failure being the main reason for this high number of deaths [1]. MI produces the death of cardiomyocytes in the affected area of the heart and dead tissue is replaced by a fibrotic scar to prevent ventricular wall rupture. In long-standing heart failure, interstitial fibrosis accumulates and leads to cardiomyopathy, progressively worsening the cardiac function. The fibrotic response results in a mechanical stiffness and an inflammatory environment that activate cardiac fibroblasts (CFs), promoting their proliferation and differentiation into myofibroblasts. CF activation can also be mediated by vascular cells or cardiomyocyte-derived exosomes, whose specific RNA or protein content play pivotal roles in this process [2,3,4,5]. These activated CFs produce and secrete more extracellular matrix (ECM) components that complexify and enlarge the fibrotic scar. CFs also express contractile genes, such as smooth muscle α-actin (α-SMA), to enable their contraction and thus their ability to close the wound [6,7].

Although the formation of the fibrotic tissue is initially beneficial, as it replaces the dead cardiomyocytes and prevents muscular wall rupture, its perpetuation and expansion lead to adverse heart remodeling [8]. Different therapies are being tested for the treatment of MI, some focusing on the induction of myocardial revascularization such as the recruitment of endothelial progenitor cells to the ischemic zone, thus contributing to the recovery of the ischemic damage and the reduction in the extent of MI and scar formation [9,10,11]. Many other therapies have also focused on directly inhibiting or reversing cardiac fibrosis, primarily by targeting the major activation signaling pathways of CFs. Unfortunately, the existing therapies for the treatment of MI are palliative and the anti-fibrotic treatments are currently considered inefficient and non-specific [12]. Identification and characterization of CFs is critical to understand the molecular mechanisms involved in CF behavior in order to develop new anti-fibrotic therapies.

It has been a challenge to define fibroblast-specific markers due to their heterogenous origin and phenotypic plasticity. In the past, CFs were identified and isolated by excluding other cell types such as endothelial cells (ECs), cardiomyocytes or vascular smooth muscle cells. Additionally, some markers such as α-SMA, discoidin domain-containing receptor 2 (DDR2), fibroblast-specific protein 1 (FSP1), stem-cell antigen 1 (Sca1) and vimentin were initially used to identify CFs; however, these were not unique markers of fibroblasts and were also not expressed by the whole CF population [13,14,15,16]. Currently, other proteins are extensively used to identify the CF population: platelet-derived growth factor receptor α (PDGFRα), collagen1a1 (Col1a1) and collagen1a2 (Col1a2), transcription factor 21 (Tcf21), periostin (Postn) and CD90, although in this latter only comprises around 60–70% of the total CF population [6,16,17,18]. mEFSK4 antibodies have also been widely used to identify these cells, as they co-stain with PDGFRα+ and Col1a1+ markers [15].

Several mouse strains have been described for the specific purpose of studying fibroblast behavior. Some of these animal models are based on a direct fibroblast reporter system in which the expression of reporter genes, such as fluorescent proteins, are under the control of fibroblast-specific promoters [19]. Two promising fibroblast reporter mouse strains have been developed: PDGFRα-GFP and Col1a1-GFP [20,21,22,23]. The indirect reporter Cre-loxP system has been widely used as well. The Cre recombinase protein triggers DNA cleavage/excision between two loxP sites that are within a gene of interest, inactivating the gene after Cre recombination. In this way, a specific gene’s biological role can be studied. A Cre reporter tool strain can be also designed to have a *loxP*-flanked STOP cassette preventing transcription of a CAG promoter-driven fluorescent protein variant, inserted into the *Gt (ROSA) 26Sor* locus. Robust cell fluorescence is achieved following Cre-mediated recombination, thus allowing cell tracking. Hence, gene expression may be temporally controlled with a ligand inducible Cre-loxP system, where an inactive fusion protein, composed of the Cre recombinase protein fused to one (Cre-ERT2) or two (MerCreMer (MCM)) mutant estrogen receptor ligand-binding domains, is expressed. Once tamoxifen is administered, the fusion protein is activated, leading to its nuclear translocation and induction of recombination of the floxed DNA [24]. 

Several transgenic mouse models developed with different Cre-driven fibroblast-specific promoters have been developed for experimental studies, such as the Col1a1 and Col1a2-CreERT2, PDGFRα-CreERT2, Tcf21-CreERT2 or MCM and PostnMCM [6,16,25,26,27]. In this study, we have performed a comparison between three of the most commonly used CF–Cre recombinase mouse lines in cardiac fibrosis (PDGFRα, Postn and Col1a1), through the Cre inducible system, to determine which one might be the best for tracing and/or manipulation of CFs. 

Our results provide data based on a comparative analysis wherein the reliability and reproducibility of Cre recombinase expression as well as the detection sensitivity of the reporter have been evaluated.

## 2. Materials and Methods

### 2.1. Animal Models 

All animal requisitions, housing, treatments and procedures were performed according to all state and institutional laws, guidelines and regulations. All studies were approved by the Ethics Committee for Animal Research at the University of Navarra and the Government of Navarra. B6.129S-*Pdgfra^tm1.1 (cre/ERT2)Blh^*/J (Pdgfr*a*-CreERT2, stock Jackson: 032770) [27], B6.29S-Postn^tm2.J (cre/Esrl*)^/J (PostnMCM, stock Jackson: 029645) [16], B6.Cg-Tg (Col1a1^cre/ERT2^)1Crm/J (Col1a1-CreERT2, stock Jackson: 016241) [25], B6.Cg-Gt (ROSA)/26Sor^tm9 (CAG-tdTomato^) (tdTomato, stock Jackson: 007909) [28] and B6.Cg-Gt (ROSA)26Sor^tm6 (CAG-ZsGreen1)Hze^/J (ZsGreen, stock Jackson: 007906) [28] mice were purchased from the Jackson Laboratory (ME, USA) and have been previously described. 

### 2.2. Study Design

To assess inducible Cre recombinase activity in CFs, reporter mice (bred in homozygosis) were crossed with fibroblast-specific inducible Cre-driver mice (bred in heterozygosis). Tamoxifen was administered to both, the offspring of this mating containing the Cre allele (Cre-positive mice) and the offspring of this mating which did not contain the Cre allele (Cre-negative mice, used as controls), as explained below (see “Tamoxifen treatment”). Cre recombinase activity was assessed with fluorescence visualization of macroscopic whole heart and microscopic sections, as well as with flow cytometry analysis (see “Heart processing” and “Flow cytometry analysis”), in healthy and MI 8-week-old mice (see “Induction of MI in mice”). The experimental schemes for tamoxifen treatment, MI induction and sacrifice time-points performed in each mouse model are detailed in the “Results” section. 

### 2.3. Tamoxifen Treatment

Tamoxifen (T5648, Sigma) was administered to Pdgfr*a*-CreERT2 mice by intraperitoneal (i.p.) injections at 100 mg/Kg of body weight during three consecutive days. One week after the last dose, MI was induced. Healthy control mice were sacrificed one week after the last dose of tamoxifen, whereas infarcted mice were sacrificed one week after MI. For induction of the PostnMCM mice, 2 or 5 i.p. injections of tamoxifen (dose 100 mg/Kg of body weight) were administered starting two days after MI injury. Healthy and MI mice were sacrificed one week after the last tamoxifen injection. For induction of the Col1a1-CreERT2 mice, 4 doses of tamoxifen (100 mg/Kg or 160 mg/Kg of body weight) were administered over 4 consecutive days starting at 1 day after MI injury. Healthy and MI mice were sacrificed one week after the last tamoxifen injection. The tamoxifen treatment procedure was followed as described in the Jax Cre Repository and as previously described in the literature [29,30]. Cardiotoxicity was not found at the administered doses of tamoxifen.

### 2.4. Induction of MI in Mice 

Myocardial infarction (MI) was induced in mice by ligation of the left anterior descending (LAD) coronary artery as previously described [23]. Briefly, 8-week-old mice were anesthetized with vaporized isoflurane, intubated using a 20 G intravenous catheter, mechanically ventilated and placed on a heating pad to maintain body temperature. A left thoracotomy was performed at the fourth–fifth intercostal space, where muscles were dissected. The LAD coronary artery was permanently ligated using a 7/0 non-absorbable ethylene suture. After visual verification of anemia and akinesis of the apex and anterior-lateral wall to ensure coronary occlusion, the thorax was closed in layers. After extubation, mice were kept warm until fully recovered. 

### 2.5. Heart Processing

Hearts from healthy or infarcted mice were isolated at the selected time points, cold phosphate-buffered saline (PBS) (Lonza)-perfused and processed for histological and cytometry analyses.

Excised organs were macroscopically examined under a fluorescence lamp (Stereo Microscope Fluorescence SMZ1000, Nikon) and pictures were taken under the same conditions for all the animals.

For histological studies, hearts were fixed with Zinc Formaline solution (Thermo Scientific) overnight at 4 °C, cryoprotected in 30% sucrose for 24 h and embedded in OCT compound (VWR), frozen in dry ice and stored until sectioning. Six micron tissue sections were nuclei counterstained with Hoechst 33258 (Invitrogen, 1 μg/mL for 5 min) and mounted in PBS:Glycerol (1:1). Images were captured with a Vectra Polaris scanner from Akoya Bioscience and edited with the Phenochart program.

Heart fibrosis was assessed in Sirius Red (SR) and Masson’s trichrome (MT) stained sections. For SR stainings, cryosections were hydrated in PBS for 5 min, immersed in 0.1% Fast Red (Sigma) in a saturated solution of picric acid for 90 min, differentiated 2 min in 0.01N HCl (Sigma) and mounted in DPX (Sigma). For MT stainings, sections were stained with Weigert’s Iron Hematoxylin, Aniline Blue and Biebrich scarlet-acid fuchsin stains, respectively, following the detailed protocol of Sridharan et al. [31]. SR and MT images were captured in an Aperio CS2 scanner from Leica Biosystems and edited with Image Scope program.

For cytometry analyses, mouse CFs were obtained from individual 8-week-old mice as previously described [23]. Briefly, after euthanasia, the thorax was opened and the heart was perfused with PBS pH 7.6, the atria were discarded and the excised ventricles placed in ice cold DMEM medium (Sigma) supplemented with 10% fetal bovine serum (FBS) (Hyclone). Ventricles were minced using a sterile scalpel. Pieces of tissue were incubated on an orbital shaker for 7 min at 37 °C in the presence of Liberase TH (125 µg/mL) (Roche) in HBSS++ solution (Hanks balanced salt solution, Gibco). After enzymatic incubation, partially digested tissue was mechanically dissociated by slowly pipetting to generate a single cell suspension. The digestion was repeated with the sedimented pieces, and the supernatants were pooled together. The total time for enzymatic digestion was 20 min. The supernatant was filtered through a cell strainer to remove cardiomyocytes (40 μm, nylon; Falcon). Erythrocytes were removed using RBC lysis buffer (eBioscience). The cell pellet was resuspended in 200 µL FACS buffer (2 mM EDTA, 0.5% BSA in PBS) and incubated with 20 µL of Feeder Removal MicroBeads (mEFSK4) (Miltenyi) for 15 min at 4 °C. A positive selection of CFs and a negative selection of endothelial and other non-fibroblast cells was performed using LS columns (Miltenyi) according to the manufacturer’s instructions. 

### 2.6. Flow Cytometry Analysis 

For flow cytometry characterization, the cell pellet was resuspended in 100 μL of sorting buffer with the anti-feeder cells antibody (mEFSK4 clone) (Miltenyi, 1:100) and CD31 antibody (Biolegend, 1:100). Unlabeled cells were used as a negative control for antibody labeling. After a 15 min incubation at R/T in the dark, samples were washed twice with FACS buffer and spun at 650 g for 5 min. Then, the supernatant was discarded, and the final pellet was resuspended in 250 μL of FACS buffer. Just before flow cytometry, 7-aminoactinomicina D (7-AAD) (Thermo Scientific) was added to each sample to determine viability. Standard, strict forward scatter width versus area criteria were used to discriminate doublets and gate only singleton cells. Viable cells were identified by staining with 7-AAD. Viable cells gated on the FSC/SCC were analyzed on the basis of the expression of mEFSK4 and CD31 antibodies as well as tdTomato/ZsGreen reporter protein expression in each subpopulation. Flow cytometry for these samples was performed on a CytoFLEXLX Flow Cytometer (Beckman Coulter, Life Sciences). The files generated by flow cytometry were processed using FlowJo Software (Tree Star, Ashland, USA). 

## 3. Results

The efficiency and specificity of different transgenic mouse models developed with fibroblast-related promoters (Pdgfr*a*, Postn and Col1a1) for inducible Cre recombinase expression were comparatively assessed. For that purpose, these mouse lines were crossed with tdTomato/ZsGreen reporter mice and fibroblast labeling was analyzed.

**Figure 1:**
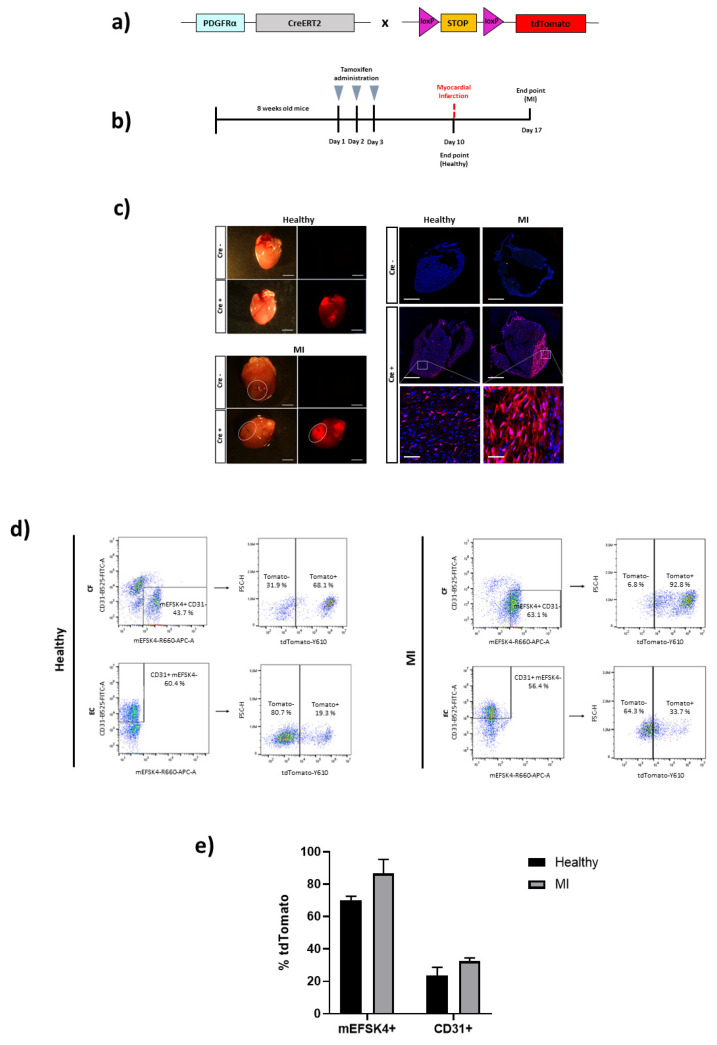
Cardiac fibroblast tracing in a Cre-inducible Pdgfr*a* mouse model of MI. (**a**) Schematic representation of the Pdgfr*a*-CreERT2 mouse crossed with a tdTomato reporter mice containing loxP sites flanking a stop codon upstream of tdTomato to allow for Cre-dependent expression. (**b**) Experimental scheme whereby 8-week-old Pdgfr*a*-CreERT2 × tdTomato mice, both healthy control and MI, were i.p. injected with 100 mg/Kg of tamoxifen for three days. One week after the last dose received, healthy control mice were sacrificed, or MI injury was induced. Infarcted mice were sacrificed one week after injury for heart analyses. (**c**) Representative images of hearts from Pdgfr*a*-CreERT2 × tdTomato mice for direct tdTomato fluorescence. Healthy control and infarcted hearts (Cre- and Cre+) are shown. Representative immunofluorescence images for tdTomato expression of Pdgfr*a*^+^ cells (red) and Hoescht staining (blue) for the nucleus. Images of healthy control and infarcted heart sections (Cre- and Cre+) with amplified images of the hearts from Cre+ mice are shown (n = 2–3 mice per condition). Scale bars: 800 μm and 50 μm (amplified images). (**d**) Representative flow cytometry plots of tdTomato^+^ cells from mEFSK4^+^/CD31^−^ (CF) or from mEFSK4^−^/CD31^+^ (EC) subpopulations of Cre+ healthy control and MI hearts (n = 2 mice per condition). (**e**) Quantification of mEFSK4^+^-tdTomato^+^ and CD31^+^-tdTomato^+^ cells in healthy control and infarcted hearts. Results represent the mean ± S.D.

A Pdgfr*a*-CreERT2 line was first evaluated for CF identification, as Pdgfr*a* has been reported to be a universal CF marker for undifferentiated fibroblasts as well as for differentiated myofibroblasts [17,32]. Therefore, the Pdgfr*a*-CreERT2 mouse line was crossed with tdTomato reporter mice (Figure 1A) and the adult offspring treated with tamoxifen (100 mg/Kg of body weight, i.p. injection) for three consecutive days. One week after the last dose, the animals were infarcted (Figure 1B) and another week after MI injury, sacrificed. A Macroscopic assessment of the hearts’ with tdTomato fluorescence revealed a high expression in the infarcted zone due to the proliferation and migration of CFs to the injured area. Fluorescence was also detected in the uninjured zone of the heart; however, this was expected because the CF abundance was lower, and it exhibited a lower intensity. tdTomato expression was also found in healthy control animals one week after tamoxifen administration, with a pattern similar to the remote zone of infarcted hearts. Histological analyses on cryosections of the hearts confirmed these results (Figure 1C). The healthy and infarcted hearts from Cre-negative animals (also tamoxifen-treated) did not show tdTomato expression.

In order to quantify the level of labelled CFs from the total population, as well as the specificity of the reporter mouse line for CF targeting, we also used flow cytometry to quantify the percentage of CFs (mEFSK4^+^/CD31^−^) and ECs (mEFSK4^−^/CD31^+^) in tdTomato-positive cells (Figure 1D,E). Cre recombination was found in a high percentage of total CFs in control animals (70.1 ± 2.4%) while it reached 86.7 ± 8.6% in the infarcted animals. It should be noted, however, that the mEFSK4 antibody, used to analyze the CF population, also stains a subpopulation of CD11b^+^ leukocytes [15], so we cannot exclude the possibility that the CF population also contains these cells in a low percentage. tdTomato expression was also found in ECs from healthy control and MI hearts, reaching 23.5 ± 5.2% and 32.7 ± 1.8%, respectively. Confirming the above histological results, tdTomato+ CFs or ECs were not detected in Cre-negative mice after tamoxifen administration (Figure S1).

**Figure 2:**
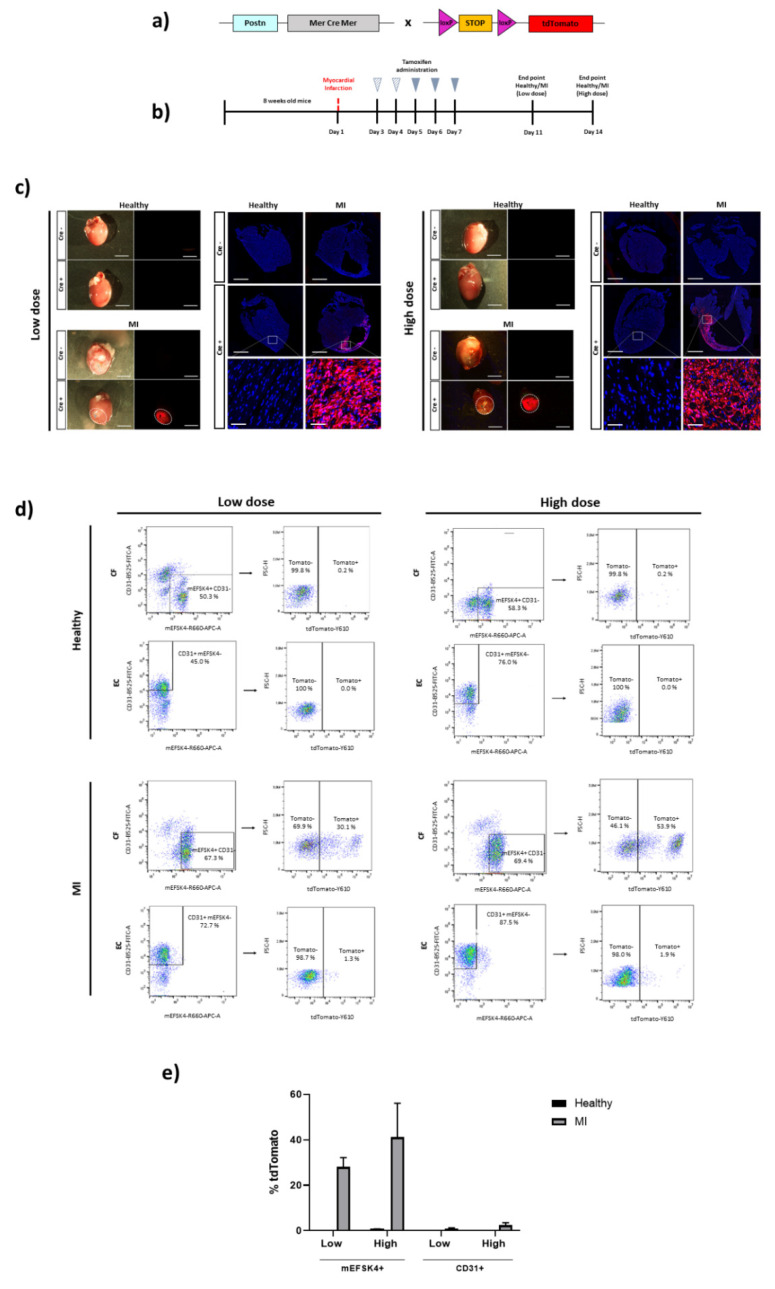
Cardiac fibroblast tracing in a Cre-inducible Postn mouse model of MI. (**a**) Schematic representation of the PostnMCM mouse crossed with a tdTomato reporter mice containing loxP sites flanking a stop codon upstream of tdTomato to allow for Cre-dependent expression. (**b**) Experimental scheme whereby 8-week-old PostnMCM x tdTomato mice were i.p. injected with 100 mg/Kg of tamoxifen after MI injury for two (low dose) or five (high dose) days. Animals were sacrificed and hearts analyzed one week after receiving the last dose. (**c**) Representative images of hearts from PostnMCM x tdTomato mice for direct tdTomato fluorescence. Healthy control and infarcted hearts (Cre- and Cre+) are shown for low and high doses. Representative immunofluorescence images for tdTomato expression of Postn+ cells (red) and Hoescht staining (blue) for the nucleus. Images of healthy control and infarcted hearts (Cre- and Cre+) are shown for low and high doses, including amplified images of the sections from Cre+ hearts (n = 2–4 mice per condition and dose). Scale bars: 800 μm and 50 μm (amplified images). (**d**) Representative flow cytometry plots of tdTomato+ cells from mEFSK4+/CD31- (CF) or from mEFSK4-/CD31+ (EC) subpopulations of Cre+ healthy control and MI hearts treated with low and high tamoxifen doses (n = 2–3 mice per condition and dose). (**e**) Quantification of mEFSK4+-tdTomato+ and CD31+-tdTomato+ cells in healthy control and infarcted hearts, treated with low or high tamoxifen doses. Results represent the mean ± S.D.

A PostnMCM line was also evaluated for CF identification. The PostnMCM mouse was crossed with tdTomato reporter mice for Cre expression analyses (Figure 2A). Postn is expressed in stromal cells during embryo development, downregulated in the adult and upregulated again after cardiac injury [33]. Taking this into account, tamoxifen induction (100 mg/Kg of body weight, i.p. injection) began two days after MI induction. Healthy control and infarcted mice were given two (low dose) or five (high dose) daily injections of tamoxifen and sacrificed one week after receiving the last dose (Figure 2B). Macroscopic and histological analyses clearly showed tdTomato expression in the infarcted hearts at both doses, whereas no positive cells were detected in healthy control animals. Again, no cell labeling was found in Cre-negative animals (Figure 2C). 

Flow cytometry analyses confirmed tdTomato expression in CFs isolated from infarcted hearts, further showing a trend to increase in the percentage of tdTomato+ CFs in animals treated with five tamoxifen doses in comparison with those treated with two tamoxifen doses (28.1 ± 4.1% (low dose) vs. 49.7 ± 4.5% (high dose)). As expected, a negligible percentage of positive stromal cells was found in healthy control animals. 

Potential Postn expression or leakage to ECs was also evaluated. tdTomato+ vascular cells were not detected in healthy control animals (0.04 ± 0.0% for low dose and 0.1 ± 0.1% for high dose). A residual percentage of positive cells was detected in infarcted hearts (1.0 ± 0.3% for low dose and 2.4 ± 1.1% for high dose) (Figure 2D,E). tdTomato+ CFs or ECs were not detected in Cre-negative mice after tamoxifen administration (Figure S2).

**Figure 3:**
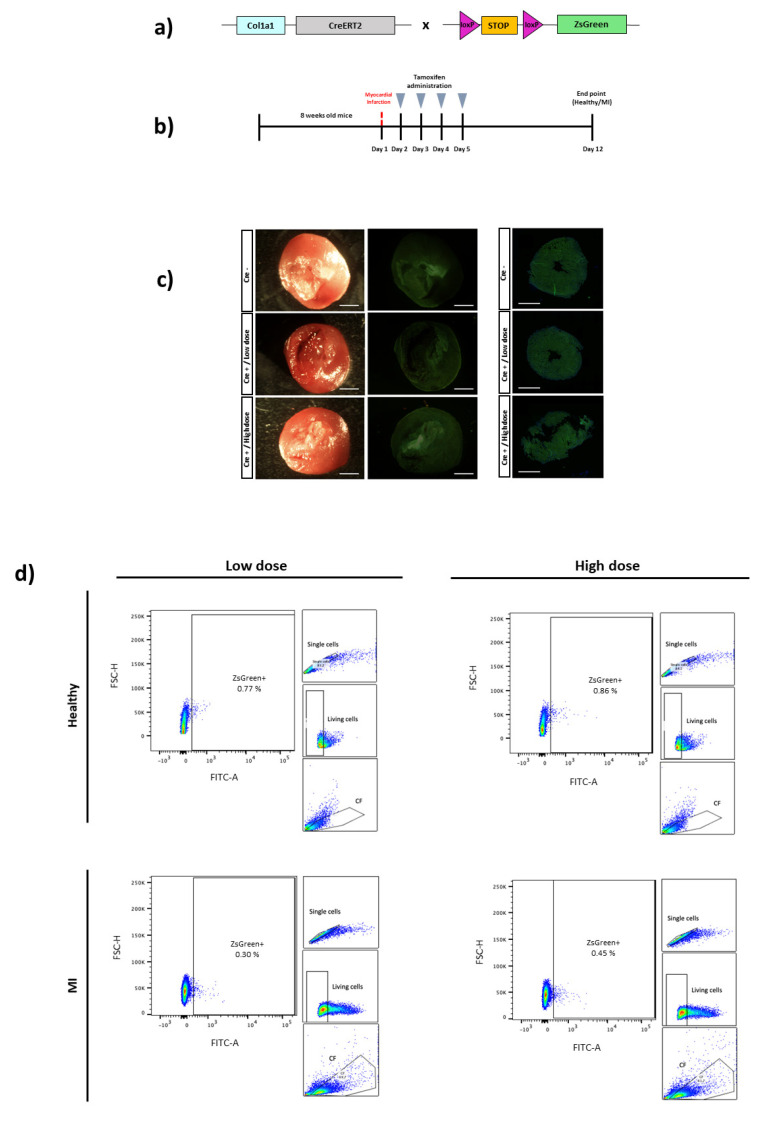
Cardiac fibroblast tracing in a Cre-inducible Col1a1 mouse model of MI. (**a**) Schematic representation of the Col1a1-CreERT2 mouse crossed with a ZsGreen reporter mice containing loxP sites flanking a stop codon upstream of ZsGreen to allow for Cre-dependent expression. (**b**) Experimental scheme whereby 8-week-old Col1a1-CreERT2 x ZsGreen mice were i.p. injected with 100 mg/Kg (low dose) or 160 mg/Kg (high dose) of tamoxifen for four days. MI mice received the first dose the day after injury. One week after the last dose received, healthy control and MI mice were sacrificed. (**c**) Representative images of hearts from Col1a1-CreERT2 x ZsGreen mice for direct ZsGreen fluorescence. Three healthy control hearts (Cre-, Cre+ low dose and Cre+ high dose) are shown. Representative immunofluorescence images for ZsGreen expression of Col1a1+ cells (green) and Hoescht staining (blue) for the nucleus. Images of three control hearts (Cre-, Cre+ low dose and Cre+ high dose) are shown (n = 1–2 mice per condition). Green background in hearts corresponds to heart tissue autofluorescence. Scale bars: 800 μm. (**d**) Representative flow cytometry plots of ZsGreen+ cells isolated from the living cell populations (ancestry gates are shown on the right of the plot) of Cre+ control and MI mice hearts, at low and high doses (n = 1–2 mice per condition and dose).

Finally, the Col1a1-CreERT2 line was also assessed for the identification of CFs. The Col1a1-CreERT2 mouse was crossed with a ZsGreen reporter mouse for Cre expression analyses (Figure 3A). Although CFs express the type I collagen protein in resting and activated states, they upregulate after activation [23,34,35], so tamoxifen injection was also performed after MI induction. Healthy control or infarcted Col1a1-CreERT2 mice were i.p. injected with tamoxifen (100 mg/Kg or 160 mg/Kg of body weight, corresponding to low or high doses, respectively) during four consecutive days starting one day after MI induction (Figure 3B). One week later, ZsGreen expression in the heart was analyzed at the macroscopic and histological levels, as well as by flow cytometry (Figure 3C,D). Cre-mediated recombination was not achieved by using a 100 mg/Kg of body weight dose of tamoxifen with less than 1% of ZsGreen expression found in control or infarcted hearts from Cre-positive mice. Treatment with an even higher dose (160 mg/Kg of body weight) did not yield a greater expression of ZsGreen (Figure 3D). ZsGreen was not detected in Cre-negative mice after tamoxifen administration (Figure S3).

As a technical control for our mouse model, ZsGreen expression was assessed in other organs, such as lung and bone, confirming a robust ZsGreen expression in both organs but not in the heart (Figure S4).

Importantly, no lethality or cardiotoxicity was found after tamoxifen injection in healthy or infarcted mice from the three transgenic lines. Abnormal heart collagen deposits and fibrotic response due to cell death were not found either in the non-injured hearts or in the non-ischemic affected areas of the infarcted hearts (Figure S5).

## 4. Discussion

Fibroblasts are cells of mesenchymal origin that are distributed in the interstitial spaces of many organs and are the principal ECM-producing cells, including vimentin, fibronectin, Postn and collagen types I, III, V and VI. These stromal cells can lead to fibrosis in different diseases, including MI [36]. Different tools based on animal models have been developed in order to target and manipulate this specific cell type. In this study, we have performed a comparative analysis of three different transgenic lines with Pdgfr*a*, Postn and Col1a1 CF Cre-driven promoters, in two different mouse models: a healthy and an infarcted model, to select the most reliable and specific mouse line for heart fibrosis analyses. 

In the Pdgfr*a*-CreERT2 animal model, high levels of tdTomato expression (up to 90%) were observed in Cre-positive mice. In healthy control animals, the fluorescent signal was scattered throughout the cardiac tissue, since Pdgfr*a* is expressed in quiescent fibroblasts [17,21], which are distributed all over the heart. In hearts one-week post-infarct, a high concentration of positive fibroblasts was, as expected, also detected in the injured zone, as CFs migrate to the damaged region in response to cardiac insult, proliferating and differentiating towards myofibroblasts. Interestingly, Pdgfr*a* expression has been shown to be increased in myofibroblasts after MI [32], which could explain the observed trend toward increased numbers of positive cells in the injured hearts compared to non-injured control hearts.

Despite the optimal results for detecting CFs with this mouse model, we found that tdTomato expression was not specific to CFs, since a ~20% expression was observed in the ECs of control hearts and a ~30% expression was found in the ECs from infarcted hearts. Although PDGFRα has been shown to be primarily expressed by interstitial fibroblasts [17], its contribution to blood vessel formation [37] and expression in adult heart vessels [15,32] has also been described. Moreover, an increased expression of pro-angiogenic growth factor receptors has been shown in pathological angiogenesis, which results in an increase in the ability of ECs to proliferate and migrate [38]. ECs activation has been previously found in the inflammatory environment produced by ischemia, which could explain the trend toward the increased Pdgfr*a* expression found in these cells after infarct.

In the PostnMCM animal model, ~40% of tdTomato expression was reached in the infarcted mice treated with the highest tamoxifen dose. Postn, a secreted ECM protein involved in cellular adhesion and the organization of collagen, is specifically produced in activated/differentiated CFs [23,39,40]. Indeed, only the infarcted region of the hearts showed red fluorescence labeling, with no positive cells detected in the remote, non-injured zone, which explains the partial detection of labeled CFs by flow cytometry. Accordingly, no or very low signal was found in the healthy hearts when analyzed by histology or flow cytometry. 

Furthermore, we confirmed the specificity of this mouse model to label CFs and not other cell types of the heart, such as ECs. Although it has been reported that human pulmonary artery ECs express Postn under hypoxic conditions [41], its production by cardiac ECs in healthy or ischemic hearts has been not found to date. Accordingly, a negligible percentage (<2%) of tdTomato-positive ECs was quantified across all conditions and doses in our study, confirming CF-specific Postn expression and therefore, the suitability of this mouse model.

The synthesis of structural collagens, such as collagen I and collagen III, is a hallmark of fibroblasts in the healthy and remodeling heart, and Col1a1-GFP reporter mice have been a robust tool for general fibroblast identification in many organs, including the heart [23]. The indirect Cre-loxP system has also been described for the detection of collagen-producing cells in the heart [30], as well as in other organs [25]. However, we failed to detect ZsGreen reporter expression in the hearts of our Col1a1-CreERT2 animal model, either in the control or in MI hearts. The use of even higher doses of tamoxifen (160 mg/Kg) than those used previously did not achieve positive results. Moreover, quantitative RT-PCR of Cre mRNA extracted from CFs of Col1a1-CreERT2 mice confirmed that very low levels of Cre recombinase mRNA transcripts (CT > 35, data not shown) are being expressed in these cells under the Col1a1 promoter of this Cre-driver mouse. However, a remarkable expression of the ZsGreen reporter protein observed in the bone or the lung confirms appropriate tamoxifen-induced Cre-recombinase activity in other organs of our mouse model. This diversity of the Col1a1 promoter activity, and consequently Cre expression across organs could be due to the positional effect of a randomly integrated transgene [42]. It is worth noting that we performed similar studies with the Col1a2-CreERT animal model (B6.Cg-Tg (Col1a2^-cre/ERT,-ALPP^)7Cpd/2J), also without positive results (data not shown). Slight variations between commercial and previously reported laboratory in-house mice strains that affect the heart´s collagen promoter regulation might account for such differences in Cre recombinase expression in CFs.

Other Cre recombinase mouse lines such as the Tcf21-CreERT2 or MCM have been rather extensively used for fibroblast tracking and regulation. During embryonic development, the heart is populated with fibroblasts derived from epicardial and ECs that express Tcf21, which is an essential transcription factor for CFs [20]. Tcf21 is still expressed in the resting fibroblasts of the adult heart and, therefore, these lines have been successfully employed to lineage-trace CFs during embryonic development and in adult cardiac tissue. As a limitation, the ability of this line to regulate gene expression in CFs has been shown to dramatically decrease after differentiation toward myofibroblasts [6]. The myofibroblast-specific expression of the target genes may be less efficient, as the condensed chromatin may reduce the accessibility of Cre to LoxP sites in the genes that are not being actively expressed. For an efficient recombination, pre-injury tamoxifen treatment is required. In contrast, the PostnMCM mouse model is very efficient in manipulating myofibroblast gene expression due to the upregulation of the Postn gene after cardiac injury [33]. Combining both cell lines may be a future strategy to reach a more efficient and complete recombination of CFs in different states.

While the Cre technology has provided the unique ability to spatio-temporally regulate gene expression in a relatively controlled manner, there are some caveats associated with this technique. Several studies have reported side effects of Cre expression in a variety of tissues. Thus, cardiac toxicity has been shown in αMHC-MCM mice after tamoxifen administration, provoking cardiomyocyte transcriptomic changes and apoptosis, focal fibrosis and acute cardiac dysfunction [43,44]. Cre recombinase itself can be toxic as its recombination at cryptic or pseudo loxP sites of the genome can cause DNA damage and consequently, cell death [45]. This toxic effect has also been reported in fibroblasts, which have exhibited growth arrest and chromosomal abnormalities [46]. However, the damage that the permanent presence of high levels of Cre recombinase induces can be minimized by its transient expression, allowing for the efficient recombination of loxP-flanked target alleles with minimal toxicity. These adverse effects are tamoxifen-dose-dependent [47]; therefore, a fine dose adjustment in the animal models is mandatory. Importantly, in our study, the tamoxifen doses were adjusted to optimally detect reporter expression, but without inducing cardiotoxicity. No lethality was caused after the injection in healthy or infarcted mice. Furthermore, abnormal heart collagen deposits and fibrotic response due to cell death were not found either in the non-injured hearts or in the non-ischemic affected areas of the infarcted hearts.

Hence, the protocols and results herein reported may be of interest for carrying out cell-tracking in vivo experiments as well as specific gene functional analyses, in order to study the cellular and molecular mechanisms responsible for the development of cardiovascular diseases. 

## 5. Conclusions

Taken together, the results presented here show a parallel comparison of three mouse lines based on the indirect reporter Cre-ERT2/MCM system to determine their reliability and specificity for CF tagging. Although in other studies the Col1a1-CreERT2 mouse model has shown positive results, we were not able to detect fluorescence-reporter expression in the heart, even by using high doses of tamoxifen. In contrast, with the Pdgfr*a*-CreERT2 model, a high expression of tdTomato was found, although it was not entirely specific to CFs, since a moderate expression of the reporter protein was also observed in ECs. In the PostnMCM mouse model, tdTomato expression was specifically detected in myofibroblasts and non-resting CFs, which could be of great interest for animal injury models. In addition, it was verified that this expression was specific to CFs and not ECs, thus making this model potentially the most reliable and specific animal model for future studies in cardiovascular disease.

## Data Availability

The data that support the findings of this study are available upon request from the corresponding author.

## Supplementary Materials

The following supporting information can be downloaded at: https://www.mdpi.com/article/10.3390/biomedicines10102350/s1, Figure S1: Flow cytometry plots of PDGFRα Cre- controls; Figure S2: Flow cytometry plots of Postn Cre- controls; Figure S3: Flow cytometry plots of Col1a1 Cre- controls; Figure S4: Col1a1-CreERT2 mouse model validation; Figure S5: Cardiotoxicity analyses in Cre-inducible mice lines.
