# Supplementary material for: Comparative Evaluation of Inducible Cre Mouse Models for Fibroblast Targeting in the Healthy and Infarcted Myocardium
Source: Biomedicines. 2022 Sep 21;10(10):2350. doi: 10.3390/biomedicines10102350 (PMC9598630; doi:10.3390/biomedicines10102350)
Supplement: Supplementary file 1 [file biomedicines-10-02350-s001.zip › biomedicines-1878600-supplementary.pdf]

Supplementary Materials:

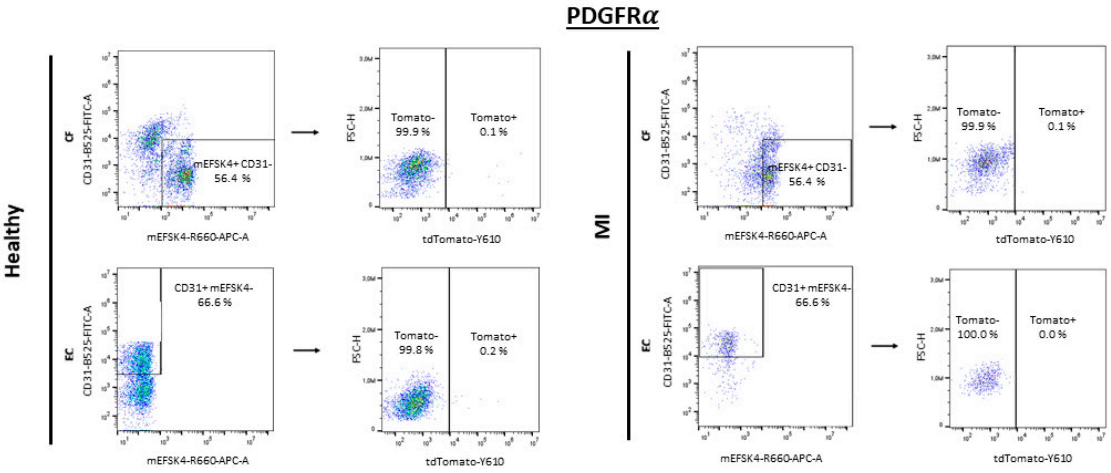

Supplementary Figure S1. Flow cytometry plots of PDGFR*α* Cre- controls. Representative flow cytometry plots of tdTomato+ cells from mEFSK4+ / CD31- (CF) or from mEFSK4- / CD31+ (EC) subpopulations of healthy control and MI hearts.

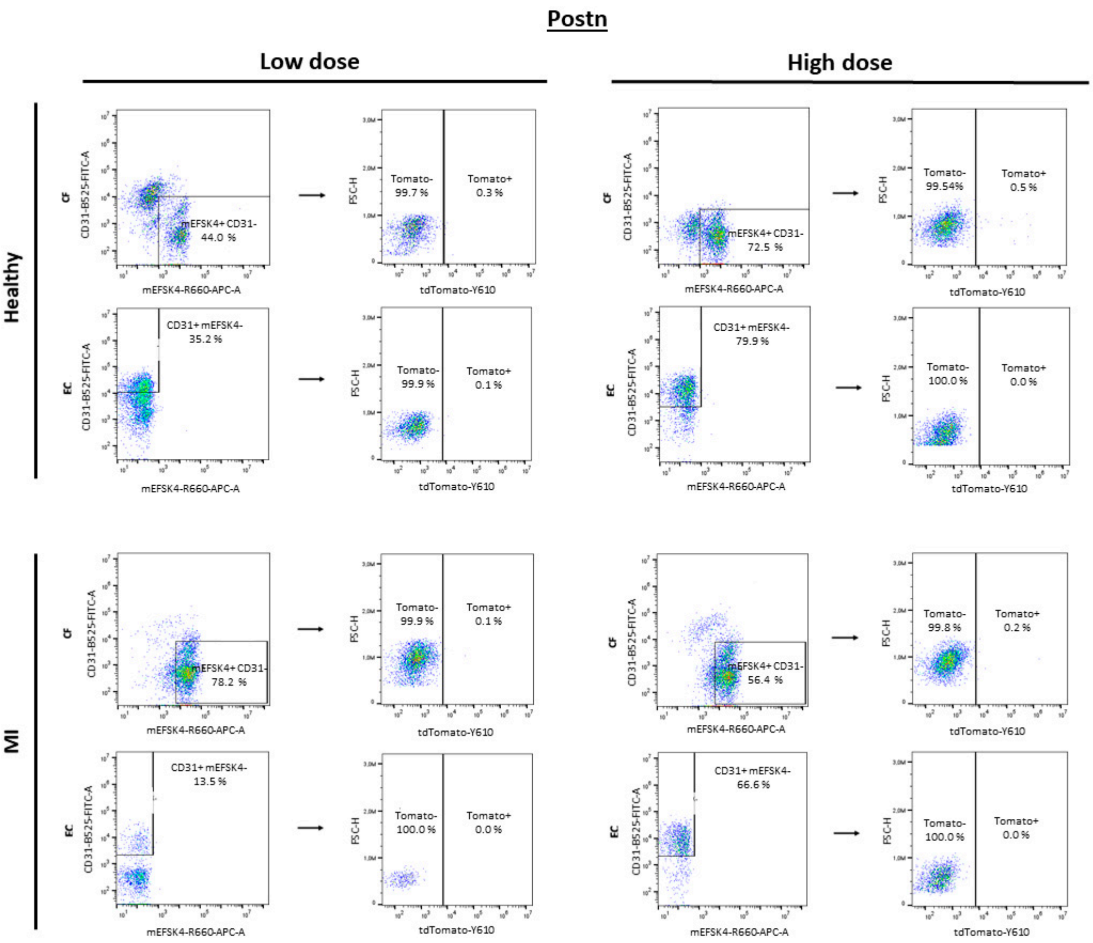

Supplementary Figure S2. Flow cytometry plots of Postn Cre- controls. Representative flow cytometry plots of tdTomato+ cells from mEFSK4+ / CD31- (CF) or from mEFSK4- / CD31+ (EC) subpopulations of healthy control and MI hearts treated with low and high tamoxifen doses.

Col1a1

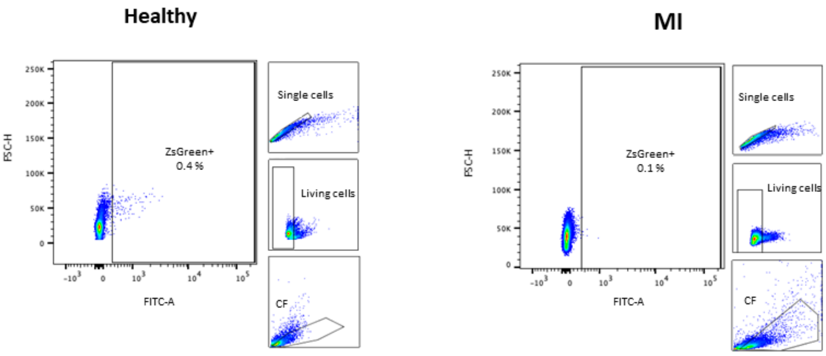

Supplementary Figure S3. Flow cytometry plots of Col1a1 Cre- controls. Representative flow cytometry plots Zsgreen+ cells isolated from the living cell populations (ancestry gates are shown on the right of the plot) of healthy and MI mice hearts.

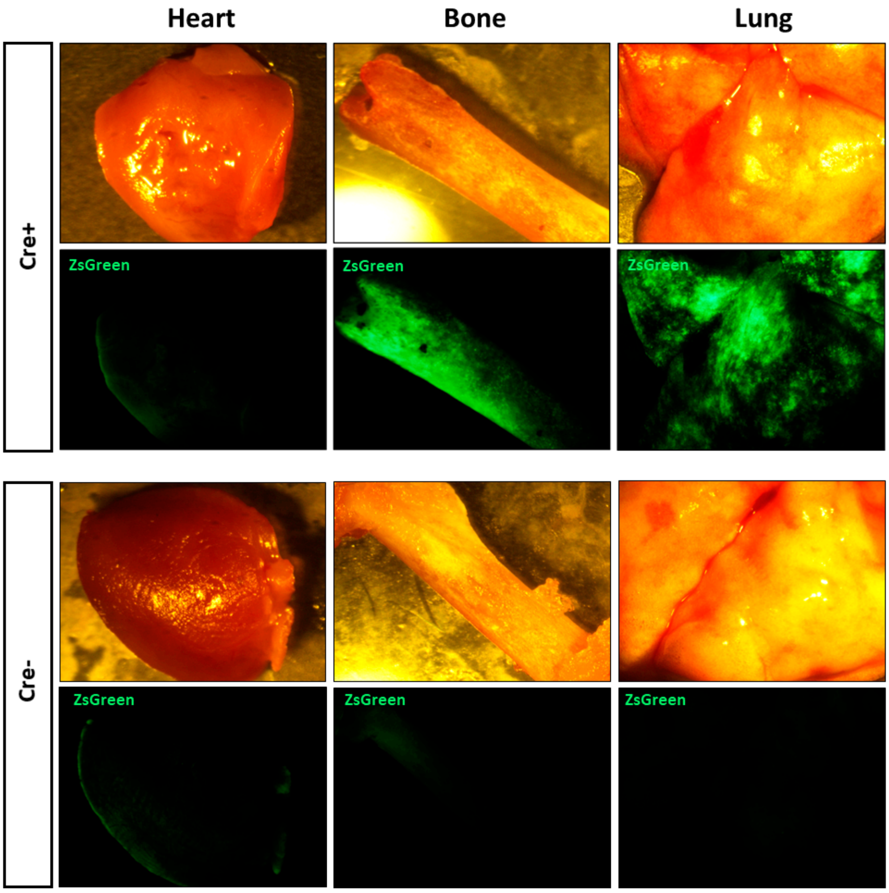

Supplementary Figure S4. Col1a1-CreERT2 mouse model validation. Representative images of the heart, bone and lung from Col1a1-CreERT2 x ZsGreen mice for direct ZsGreen fluorescence. High dose tamoxifen treated Cre+ and Cre- control mice are shown.

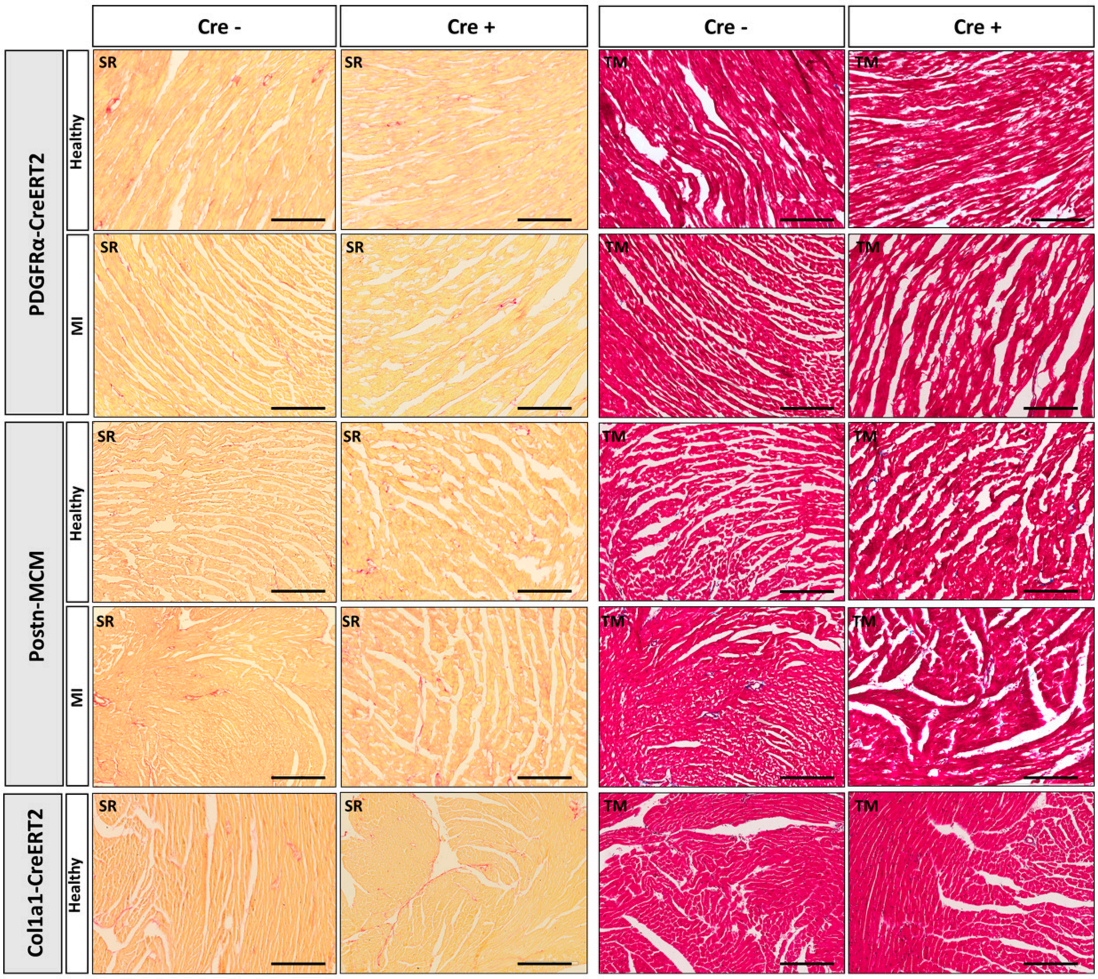

Supplementary Figure S5. Cardiotoxicity analyses in Cre-inducible mice lines. Representative images of Sirius red and Masson´s trichrome heart-sections from Pdgfra-CreERT2, PostMCM and Col1a1-CreERT2 Cre+ and Cre- tamoxifen-treated mice (healthy and MI) are shown. Pictures show the remote area of the infarcted hearts. Scale bars: 200µm.
